# Ants of the Palouse Prairie: diversity and species composition in an endangered grassland

**DOI:** 10.3897/BDJ.9.e65768

**Published:** 2021-05-10

**Authors:** Kayla A Dilworth, Marek L Borowiec, Abigail L Cohen, Gabrielle S Mickelson, Elisabeth C Oeller, David W Crowder, Robert E Clark

**Affiliations:** 1 Washington State University, Pullman, United States of America Washington State University Pullman United States of America; 2 University of Idaho, Moscow, United States of America University of Idaho Moscow United States of America

**Keywords:** Palouse Prairie, ant biodiversity, rapid species inventory, agroecosystems, insect conservation, prairies

## Abstract

Grasslands are globally imperilled ecosystems due to widespread conversion to agriculture and there is a concerted effort to catalogue arthropod diversity in grasslands to guide conservation decisions. The Palouse Prairie is one such endangered grassland; a mid-elevation habitat found in Washington and Idaho, United States. Ants (Formicidae) are useful indicators of biodiversity and historical ecological disturbance, but there has been no structured sampling of ants in the Palouse Prairie. To fill this gap, we employed a rapid inventory sampling approach using pitfall traps to capture peak ant activity in five habitat fragments. We complemented our survey with a systemic review of field studies for the ant species found in Palouse Prairie. Our field inventory yielded 17 ant species across 10 genera and our models estimate the total ant species pool to be 27. The highest ant diversity was found in an actively-managed ecological trust in Latah County, Idaho, suggesting that restoration efforts may increase biodiversity. We also report two rarely-collected ants in the Pacific Northwest and a microgyne that may represent an undescribed species related to *Brachymyrmex depilis*. Our score-counting review revealed that grassland ants in Palouse Prairie have rarely been studied previously and that more ant surveys in temperate grasslands have lagged behind sampling efforts of other global biomes.

## Introduction

Temperate grasslands and savannah are amongst the most endangered biomes in the world, having the highest rate of conversion to agriculture and the lowest rate of government protection (Hoekstra et al. 2005). In the United States, grasslands, savannah and barrens communities are critically endangered, experiencing > 98% decline in areas since European settlement (Noss and Scott 1995). Since organisms found in prairies are threatened by habitat loss, biodiversity surveys that describe the native and non-native fauna are necessary to help provide information for future conservation efforts and guide restoration to areas subject to impacts. Ants are useful biodiversity indicators in grasslands and characterising ant community composition through rapid assessment is pertinent for monitoring and evaluating global grassland restoration efforts (Peters et al. 2016).

Biodiversity surveys play a strategic role in grassland conservation, aiding arguments that habitats contain rare or endemic species (Van Schalkwyk et al. 2019). Temperate grasslands are host to a wide range of endemic plant and animal communities, including many insects of conservation concern (Sampson and Knopf 1994). A comprehensive list of globally-threatened species, the Red List, counts over 2,000 grassland species that are critically engendered, endangered or vulnerable worldwide, 108 of which are in North America (I.U.C.N. 2020). Grasslands are frequently fragmented by agricultural production and they are, therefore, prone to increased rates of local extinctions through a variety of modes, such as reduced population sizes, increased invaders and elimination of keystone predators (Leach and Givnish 1996).

The Palouse Prairie is an endangered grassland ecosystem that originally encompassed south-eastern Washington State and neighbouring northern Idaho (Looney and Eigenbrode 2012, Noss and Scott 1995). The Palouse landscape is characterised by rolling hills formed from wind-blown fertile loess soils, which form the foundation for plant communities of caespitose grass, co-dominant shrubs and forbs (Looney et al. 2009, Daubenmire 1970). The Palouse Prairie has experienced an estimated 99.9% decline in habitat across its former range due to agricultural land use, making it one of the most imperilled ecosystems in the United States (Donovan et al. 2009, Black et al. 1998). What habitat remains is fragmented into small, narrow strips of land and a majority of these fragments are smaller than two hectares with high perimeter-area ratios (Yates et al. 2004). Encouragingly, these fragments are capable of supporting several endangered species (Looney and Eigenbrode 2012).

Published biodiversity surveys are limited for the Palouse Prairie, encompassing a survey of bumblebees (Hatten et al. 2013), macromoths (Thompson et al. 2014) and forbs (Donovan et al. 2009). In this study, we chose ants (Formicidae) for the biodiversity survey in Palouse Prairie. Ants are used prominently for monitoring because they are ubiquitous in terrestrial ecosystems, sampling is relatively inexpensive and many ant species respond quickly to disturbances (Tiede et al. 2017, Hoffmann 2010, Underwood and Fisher 2006, Folgarait 1998, Andersen 1990). However, in poorly-surveyed regions, using ants as biodiversity indicators can be challenging if taxonomic resources are not well developed (Hevia et al. 2016). The Pacific Northwest Region is generally under-sampled for ants and thus up-to-date taxonomic resources are limited (Hoey-Chamberlain et al. 2010). Consequently, we included a systematic review of all ant species found and referred directly with taxonomic experts to verify all species-level identifications.

## Material and methods

### Rapid inventory survey

**Figure 1.**
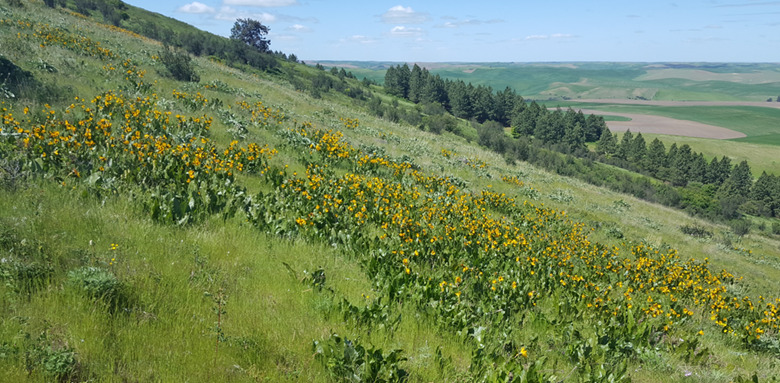
Example of an intact Palouse Prairie habitat fragment located at the Hudson Biological Preserve (“Smoot Hill”) in Albion, Washington.

**Figure 2.**
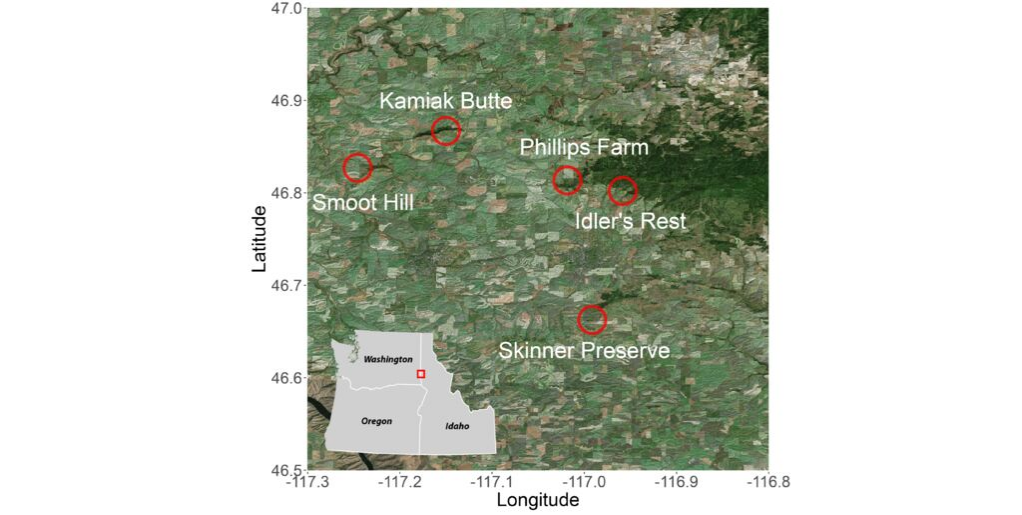
Map of survey locations for the five Palouse Prairie sites. Red circles encompass the prairie fragment sampled. Inset shows map extent in the Pacific Northwest.

Ant surveys were completed at five prairie fragments: Hudson Biological Reserve, Kamiak Butte County Park, Skinner Ecological Preserve, Idler’s Rest Nature Preserve and Philips Farm County Park (Figs 1, 2, Suppl. material 1). We used a rapid inventory sampling method with pitfall trap transects amongst the five sites (Agosti et al. 2000, Ellison et al. 2007). We used 4 cm diameter, 14 cm depth, pitfall traps filled 2/3 with propylene glycol (Higgins and Lindgren 2012). Our survey intended to capture peak ant activity in August on days above 24°C with no precipitation. We worked in four teams to ensure all traps were placed and collected simultaneously. Pitfall traps were run for 48 h, after which ants were transferred to 95% ethanol. Afterwards, ants were pinned for later identification and storage. In total, we ran 131 pitfall traps and collected 424 individual ants (Suppl. material 2).

Species-level identification of ants can be time-consuming and difficult in regions with poorly-described ant fauna (reviewed in Ellison 2012). The inland North-western US has few recent records of ants to aid in identification and we thus employed a four-step identification approach. First, all ants were identified to genus using Ant Genera of North America (Fisher and Cover 2007). Second, we used records and pinned specimen photographs posted to online ant taxonomy databases (i.e. Guénard et al. 2017) to identify ants to species. Third, we compared specimens to collections at the Washington State University MT James Museum and the University of Idaho WF Barr Entomological Museum. We also consulted published checklists of ants known from Idaho, Washington and the western United States (Cole 1936, Smith 1941, Yensen et al. 1977, Cook 1953, Wheeler and Wheeler 1986), although these studies reflect the lack of recent comprehensive reports published.

### Systematic review methods

We searched for studies in Web of Science that quantified abundance or richness of the ant species within prairie or grassland from 2016 to the present. Our search was conducted in June 2020 using the term “ant AND divers* AND prairie OR ant AND divers* AND grassland”. Our search yielded 106 studies that were reviewed for inclusion, based on three criteria: (i) the study assessed more than one species; (ii) the study was performed in non-agricultural grassland or prairie; and (iii) the biome of the study could be determined; 21 studies met these criteria (Suppl. material 3). We classified the biome of each study using either the site coordinates or other geographic descriptors to locate study areas, then cross-referenced with the EcoRegions web app (Dinerstein et al. 2017).

We examined the frequency of recent publications on each individual species collected in our pitfall survey using the same approach as the biome survey, but instead used the genus and species names as search terms. We tabulated the number of studies from 2010-2020 that reported ant species we found. We read abstracts to ensure that all studies included in this survey involved field observations of the insect species in question and we included multiple species names for recently-revised species (Schär et al. 2018). We did not include purely lab-based studies on behaviour (i.e. Bordoni et al. 2019).

### Statistical methods

Analyses and figure generation were completed in R ver 4.0.2 (CRAN 2020). Visualisation of sites was mapped using ggpmap package (Kahle and Wickham 2013). Species accumulation curves were run, using the ‘vegan’ package in R (Dixon 2003). We estimated species richness using the Chao1 estimate, an abundance-based estimate of species richness, following decision-tree recommendations (Hortal et al. 2006).

## Results

**Figure 3.**
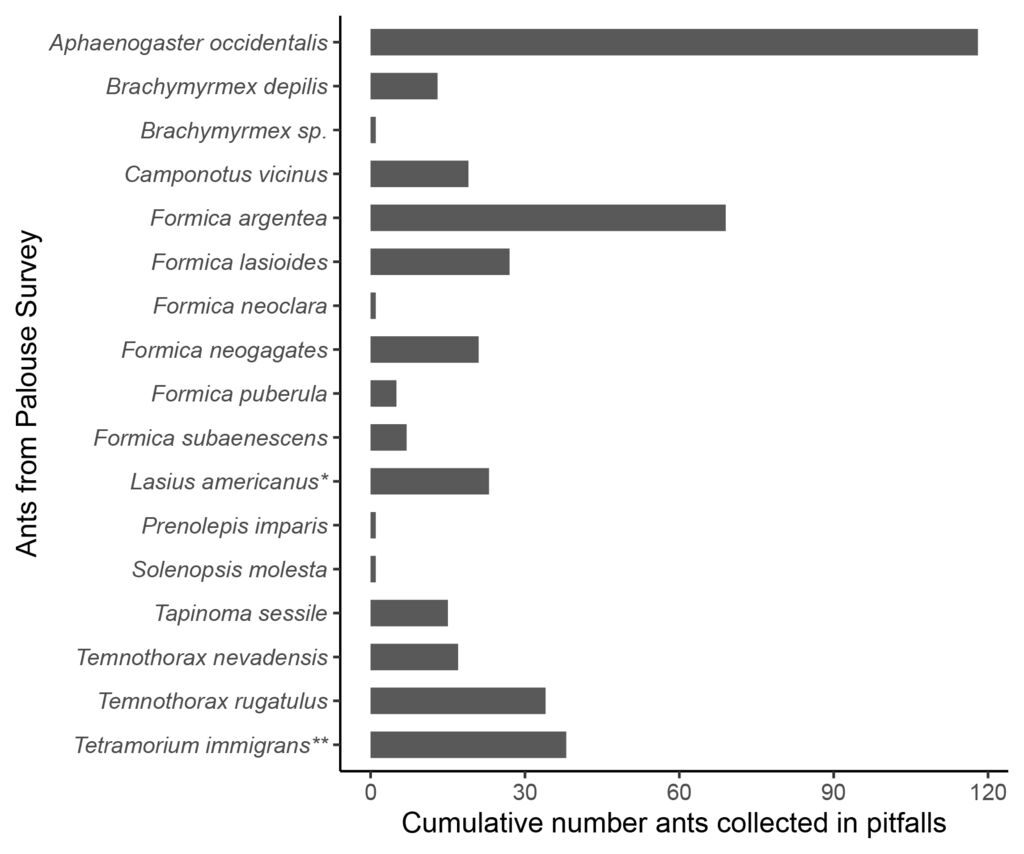
Numbers of ants collected in all pitfalls for each species. Asterisks indicates a species that has recently been revised, thus the literature search includes *Lasius neoniger and **Tetramorium caespitum. Brachymyrmex sp. indicates abundance of the *Brachyrmex* sp. microgyne collected.

Amongst all sites, we collected 17 ant species (Table 1), with *Aphaenogaster occidentalis* the most common. Ants in the genus *Formica* were the most diverse, with six species (Fig. 3). The non-native species *Tetramorium immigrans* was found in three locations, but was not abundant. We found a single microgyne (miniature queen) similar to *Brachymyrmex depilis.* Scattered records of similar *Brachymyrmex* microgynes exist and they have been interpreted as undescribed, socially parasitic species (Moreau et al. 2014, Deyrup 2016). Finally, we collected *Temnothorax nevadensis* and *Formica puberula*, two ant species that are rarely collected in Pacific Northwest temperate ecosystems (Guénard et al. 2017).

**Figure 4.**
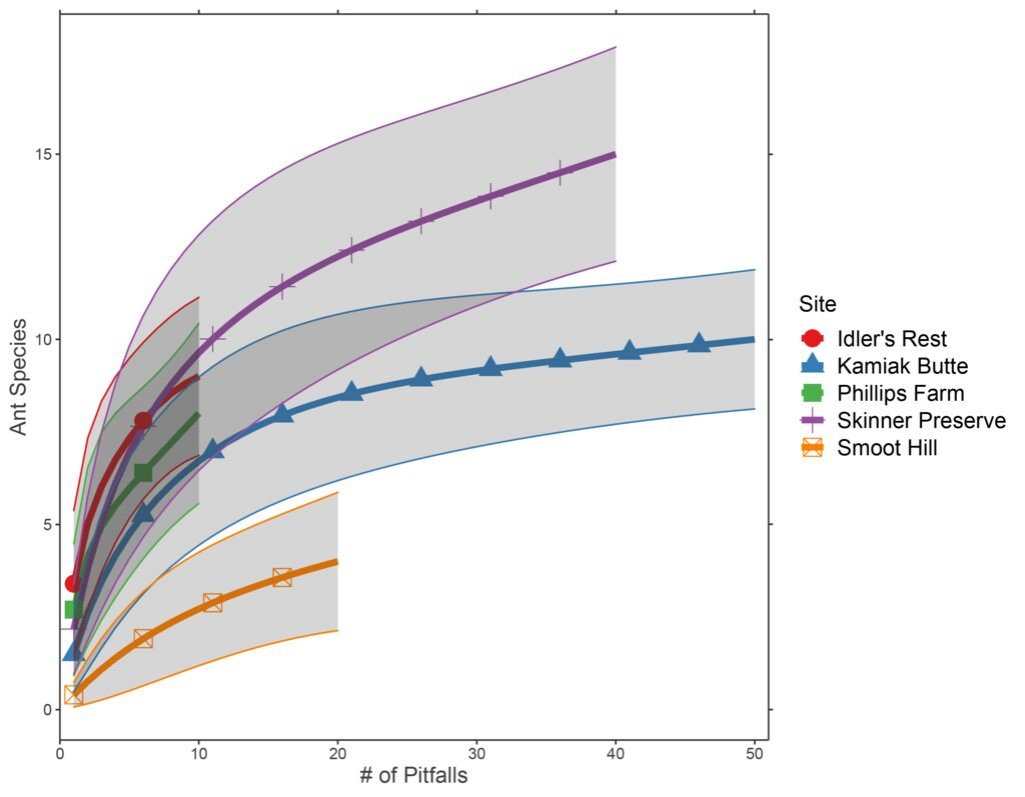
Species accumulation curve with expected mean species richness plotted for each survey location. Dots and lines show estimated species richness at a given sampling interval, while shaded area shows 95% CI.

Ant species richness varied amongst sites, with the highest diversity at Skinner Ecological Preserve (Fig. 4) and a total predicted species pool of 27 (*Chao1* index = 27.9, SE = 10.2). Skinner Preserve, the largest intact prairie fragment, had the highest species richness (16) and estimated species pool (*Chao1* index = 25.8, SE = 10.0). Smoot Hill had the lowest species richness (4) and smallest species pool (*Chao1* index = 5.9, SE = 3.6). Two restored habitats adjacent to Ponderosa Pine – Douglas-Fir forests near Moscow, Idaho had intermediate species pools (Philips Farm [9 species] – *Chao1* index = 18.0, SE = 9.2; Idler’s Rest [13.6 species] – *Chao1* index = 13.6; SE = 4.8). Kamiak Butte had 11 species (*Chao1* index = 13.9; SE = 4.4).

**Figure 5.**
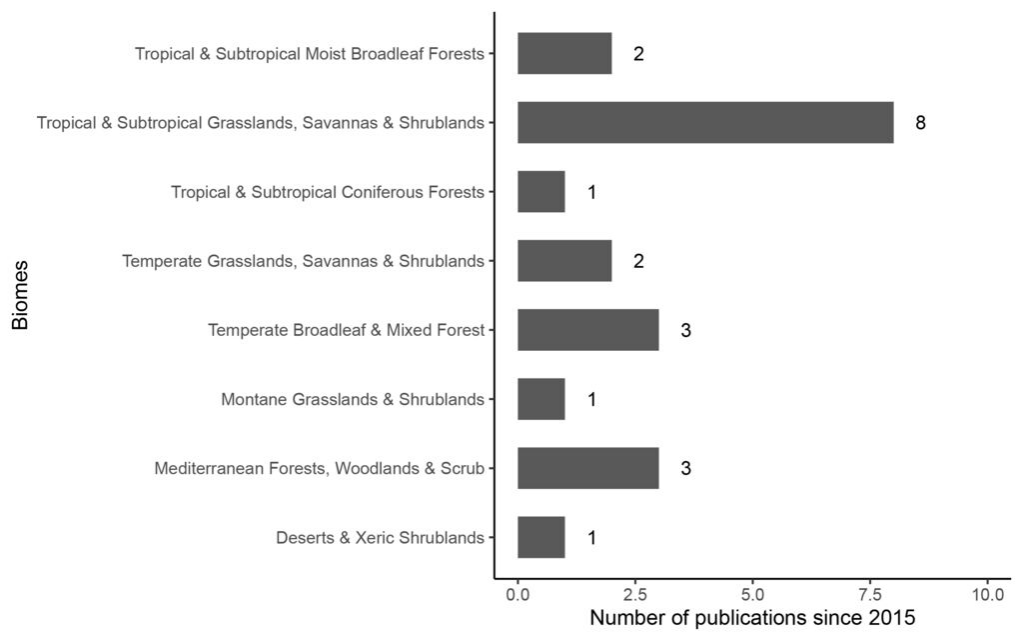
Number of ant biodiversity surveys published over a 5-year period from 2016-2020 where locations were reported. Biome classifications inferred from a global terrestrial ecoregion map (Dinerstein et al. 2017).

We found 95 publications detailing a study containing at least one species observed in our survey (Table 1). No field studies have been published for *Formica neoclara*, *F. puberula* and *F. subaenescens* and only one study included *Temnothorax nevadensis* (Table 1). These limited numbers show our pitfall sampling found several rarely studied or unstudied ant species. In fact, there were five or fewer publications for all the ant species collected besides the common “tramp species” or urban pests, like *Tapinoma sessile* and *Tetramorium immigrans* (Kamura et al. 2007, Uno et al. 2010). Our systematic review of ant biodiversity surveys in different biomes over the last five years revealed a bias towards Tropical & Subtropical Grasslands, Savannahs & Shrublands (eight studies, Cross et al. 2016, Van Schalkwyk et al. 2019, Arcoverde et al. 2016, Lasmar et al. 2020, Dröse et al. 2019, Hlongwane et al. 2019, de Queiroz et al. 2020, Santoandré et al. 2019). We found two studies on Tropical & Subtropical Moist Broadleaf Forests (Lawes et al. 2017, Klunk et al. 2018), one study on Tropical & Subtropical Coniferous Forests (Cuautle et al. 2016), three studies on Temperate Broadleaf & Mixed Forests (Braschler and Baur 2016, Helms et al. 2020, Heuss et al. 2019), one study on Montaine Grasslands & Shrublands (Jamison et al. 2016), three studies on Mediterranean Forests, Woodlands & Scrub (Adams et al. 2018, Catarinue et al. 2018, Flores et al. 2018) and one study on Deserts & Xeric Shrublands (Álvarez and Ojeda 2019). In the last five years, only two citations have included information on ants in Temperate Grasslands, Savannahs and Grassland biomes (Ramos et al. 2018, Kim et al. 2018), the biome that encompasses Palouse Prairie (Fig. 5).

## Discussion

Our model results suggest there are likely many more ant species to be discovered with more intensive sampling in Palouse Prairie and that there is likely to be appreciable diversity of other insect species beyond those of the limited taxonomic focus of this study. Furthermore, one of our collected specimens may be a currently undescribed species of ant, but this putative ant species is rarely collected and taxonomic revision of *Brachymyrmex* is required to demonstrate if this is the case. In many ecosystems, insect faunas are poorly described (Berenbaum 2008, Dunn 2005). A lack of recent publications on temperate grassland ant faunas and dearth of work on several species collected in Palouse Prairie underscore the importance of survey work in endangered ecosystems. As habitat destruction and fragmentation continue, we may never be able to sample or study the more rarely-collected species (Dunn 2005, Noss and Scott 1995).

Ant communities are under-sampled in cool-temperate ecosystems compared to the tropics and sub-tropics, including temperate grasslands in the north-western United States (Ellison 2012, Radtke et al. 2014). Since ants are excellent biological indicators of ecosystem health, sampling efforts may use our data as a comparison point to see if restoration efforts have been successful (Folgarait 1998, Williams 1994). Luckily, once taxonomic resources are available, assessment of an ecosystem’s ant communities can be completed quickly with greater accuracy of species-level identification (Ellison et al. 2007). The intermediate levels of ant diversity at sites adjacent to forest validate predictions that Palouse Prairie-forest ecotones may support high biodiversity (Morgan et al. 2020) Finally, our estimates of a species pool of 27 reflect similar scales of ant species richness found in large ant surveys in grassland systems, such as Wisconsin, USA tallgrass prairie (29 species in control sites, Kim et al. 2018) and Argentinian grasslands (46 species in grassland sites, Santoandré et al. 2019). However, given our absolute species richness was 17, there is more sampling to be completed to comprehensively describe this fauna. In fact, follow up hand-collect events at Skinner Preserve completed in 2019 found five additional ant species, including *Formica altipetens*, *Formica aserva*, *Formica obscuripes*, *Formica ravida* and *Lasius interjectus* (Borowiec, unpublished data).

Research in conservation biological control has shown the value of ants as predators in agroecosystems (Way and Khoo 1992). In our study, we found multiple species of ants in genera often implicated as predators of chewing herbivores, such as *Formica* and *Camponotus* (Drummond and Choate 2011). More recent work demonstrates that social insects, including ground-nesting ants, have positive effects on dryland crop yield by increasing water and micronutrient availability to cereals (Evans et al. 2011). However, the value of these ecosystem services likely pales in comparison to the benefit ants could provide as predators of weed seeds (e.g. Evans and Gleeson 2016). Weeds and herbicide resistance are amongst the most economically-challenging pest problems in dryland agriculture (Powell and Shaner 2001) and ants have been implicated in regulating seed banks in other grasslands (Motze et al. 2013). Recovery of more Palouse Prairie could promote higher ant diversity and abundance (Lawes et al. 2017), thus increasing the likelihood that ants are available to provide these critical ecosystem services, including weed seed predation.

## Conclusions

The remaining habitat in the Palouse is highly fragmented and only protected by a range of public and private trusts (Looney and Eigenbrode 2012). This is problematic since we found several species of ants that are uncommonly collected. While these species are not endemic to Palouse Prairie, our observations suggest that opportunities to study these insects in grassland habitats are limited. Furthermore, in addition to fragmentation and disturbance from agriculture, several of our sites are invaded by the pavement ant (*Tetramorium immigrans*), which is associated with disturbed, agricultural habitats (Cerdá et al. 2009). The extremely reduced range of the Palouse Prairie and the presence of invasive species means many other taxonomic groups of animals are in urgent need of sampling before opportunities to describe this fauna are irreversibly lost.

## Supplementary Material

9B463F24-9878-581B-AFE5-C72A8FF5E893 10.3897/BDJ.9.e65768.suppl1 Supplementary material 1 Figure 2 metadata Data type Figure metadata File: oo_514441.csv https://binary.pensoft.net/file/514441 Clark, R.E.

700F5FE3-3910-506F-91BD-8A9592EBA219 10.3897/BDJ.9.e65768.suppl2 Supplementary material 2 Figures 3 and 4 metadata Data type Figure metadata File: oo_514440.csv https://binary.pensoft.net/file/514440 Clark, R.E.

07E48EAD-EC3E-5B72-B364-BC9F4C72C2E3 10.3897/BDJ.9.e65768.suppl3 Supplementary material 3 Figure 5 metadata Data type Figure metadata File: oo_514439.csv https://binary.pensoft.net/file/514439 Clark, R.E.

## Figures and Tables

**Table 1.** Studies reporting species found in this survey between, published 2010 and 2020.

Ant species	Studies	Paper References
*Aphaenogaster occidentalis*	1	Thomas et al. 2020
*Brachymyrmex depilis*	3	King and Tschinkel 2016, Rosas-Mejía et al. 2019, Underwood and Christian 2009
*Brachymyrmex* sp. microgyne	2	Moreau et al. 2014, Deyrup 2016
*Camponotus vicinus*	1	Davenport et al. 2013
*Formica argentea*	2	Lenz et al. 2013, Ouellette et al. 2010
*Formica lasioides*	1	Karban et al. 2015
*Formica neoclara*	0	
*Formica neogagates*	5	Clark and Singer 2018, Ellison et al. 2007, Wang et al. 2001, Weseloh 2000, Weseloh 2001
*Formica puberula*	0	
*Formica subaenescens*	0	
*Lasius americanus*, reported as *Lasius alienus* in older works	11	Akyürek et al. 2016, Barton and Ives 2014, Cuesta-Segura et al. 2012, Molnár et al. 2018, Paluh et al. 2015, Park and Moon 2020, Pech 2014, Warren et al. 2012, Yitbarek et al. 2011, Zhu and Wang 2018
*Prenolepis imparis*	13	Abbate and Campbell 2013, Burford et al. 2018, Campbell et al. 2019, Cuautle et al. 2016, Fitzgerald and Gordon 2012, Gordon and Heller 2014, Kjar and Park 2016, Rowles and Silverman 2009, Sánchez-Peña 2013, Sorrells et al. 2011, Taylor et al. 2013, Thomas et al. 2020, Vonshak and Gordon 2015
*Solenopsis molesta*	4	Guyer et al. 2018, Pećarević et al. 2010, Resasco et al. 2014, Rojas et al. 2014
*Tapinoma sessile*	20	Buczkowski and Bennett 2009, Buczkowski 2010, Buczkowski and Krushelnycky 2012, Buczkowski and Richmond 2012, Gibson et al. 2019, Gow et al. 2013, Hamm 2010, Kaspari et al. 2010, Kautz et al. 2017, Kimball 2016a, Kimball 2016b, Nelson et al. 2020, Neumann and Pinter-Wollman 2019, Ouellette et al. 2010, Powell et al. 2009, Salyer et al. 2014, Schmidt et al. 2012, Scholes and Suarez 2009, Toennisson et al. 2011, VanWeelden et al. 2015
*Temnothorax nevadensis*	1	Adams et al. 2018
*Temnothorax rugatulus*	11	Bowens et al. 2013, Blonder and Dornhaus 2011, Cao 2013, Charbonneau et al. 2015, Charbonneau et al. 2017, DiRienzo and Dornhaus 2017, Doering and Pratt 2016, Doering and Pratt 2019, Doering et al. 2020, Doering et al. 2019, Heinze and Rueppell 2014
*Tetramorium immigrans* and *Tetramorium caespitum*	23	Brandt et al. 2010, Cerdá et al. 2009, Chin and Bennett 2018, Collignon and Detrain 2010, Cordonnier et al. 2019a, Cordonnier et al. 2020a, Cordonnier et al. 2020b, Cordonnier et al. 2019b, Dash and Sanchez 2009, Helms et al. 2020, Hoey-Chamberlain et al. 2010, Joharchi et al. 2012a, Joharchi et al. 2012b, Li et al. 2019, Lundgren et al. 2010, Pećarević et al. 2010, Sheard et al. 2020, Ślipiński et al. 2012, Steiner et al. 2010, Wagner et al. 2017, Wagner et al. 2018a, Wagner et al. 2018b, Joharchi and Halliday 2013
